# Gut Inflammation Markers, Diet, and Risk of Islet Autoimmunity in Finnish Children – A Nested Case-Control Study

**DOI:** 10.1016/j.tjnut.2024.05.015

**Published:** 2024-05-23

**Authors:** Tuuli EI Salo, Leena Hakola, Sari Niinistö, Hanna-Mari Takkinen, Suvi Ahonen, Leena Puustinen, Jorma Ilonen, Jorma Toppari, Riitta Veijola, Heikki Hyöty, Mikael Knip, Suvi M Virtanen

**Affiliations:** 1 Department of Public Health and Welfare, Finnish Institute for Health and Welfare, Helsinki, Finland; 2 Unit of Health Sciences, Faculty of Social Sciences, Tampere University, Tampere, Finland; 3 Tampere University Hospital, Wellbeing Services County of Pirkanmaa, Tampere, Finland; 4 Department of Virology, Faculty of Medicine and Health Technology, Tampere University, Tampere, Finland; 5 Immunogenetics Laboratory, Institute of Biomedicine, University of Turku, Turku, Finland; 6 Research Centre for Integrative Physiology and Pharmacology, Institute of Biomedicine, and Centre for Population Health Research, University of Turku, Turku, Finland; 7 Department of Pediatrics, Turku University Hospital, Turku, Finland; 8 Department of Pediatrics, PEDEGO Research Unit, Medical Research Center Oulu, University of Oulu and Oulu University Hospital, Oulu, Finland; 9 Department of Children and Adolescents, Oulu University Hospital, Oulu, Finland; 10 Fimlab laboratories, Tampere, Finland; 11 Pediatric Research Center, Children’s Hospital, University of Helsinki and Helsinki University Hospital, Helsinki, Finland; 12 Research Program for Clinical and Molecular Metabolism, Faculty of Medicine, University of Helsinki, Helsinki, Finland; 13 Department of Pediatrics, Tampere University Hospital, Tampere, Finland; 14 Center for Child Health Research, Tampere University and Tampere University Hospital, Tampere, Finland

**Keywords:** calprotectin, human β-defensin-2, gut inflammation, islet autoimmunity, diet

## Abstract

**Background:**

Gut dysbiosis and increased intestinal permeability have been reported to precede type 1 diabetes-related autoimmunity. The role of gut inflammation in autoimmunity is not understood.

**Objectives:**

This study aimed to assess whether gut inflammation markers are associated with risk of islet autoimmunity and whether diet is associated with gut inflammation markers.

**Methods:**

A nested case-control sample of 75 case children with islet autoimmunity and 88 control children was acquired from the Finnish Type 1 Diabetes Prediction and Prevention cohort. Diet was assessed with 3-d food records, and calprotectin and human β-defensin-2 (HBD-2) were analyzed from stool samples at 6 and 12 mo of age. Conditional logistic regression analysis was used in a matched case-control setting to assess risk of autoimmunity. Analysis of variance, independent samples *t* test, and a general linear model were used in secondary analyses to test associations of background characteristics and dietary factors with inflammation markers.

**Results:**

In unadjusted analyses, calprotectin was not associated with risk of islet autoimmunity, whereas HBD-2 in the middle (odds ratio [OR]: 3.23; 95% confidence interval [CI]: 1.03, 10.08) or highest tertile (OR: 3.02; 95% CI: 1.05, 8.69) in comparison to the lowest at 12 mo of age showed borderline association (*P*-trend = 0.063) with higher risk of islet autoimmunity. Excluding children with cow milk allergy in sensitivity analyses strengthened the association of HBD-2 with islet autoimmunity, whereas adjusting for dietary factors and maternal education weakened it. At age 12 mo, higher fat intake was associated with higher HBD-2 (β: 0.219; 95% CI: 0.110, 0.328) and higher intake of dietary fiber (β: −0.294; 95% CI: −0.510, −0.078), magnesium (β: −0.036; 95% CI: −0.059, −0.014), and potassium (β: −0.003; 95% CI: −0.005, −0.001) with lower HBD-2.

**Conclusions:**

Higher HBD-2 in infancy may be associated with higher risk of islet autoimmunity. Dietary factors play a role in gut inflammatory status.

## Introduction

Type 1 diabetes is an autoimmune disease already causing considerable global burden of disease and predicted to further increase in prevalence [1,2]. Type 1 diabetes is considered to be caused by a combination of genetic susceptibility and environmental triggers [2,3], but the triggers and mechanisms are not recognized well enough to enable disease prevention. Dysbiosis of the gut microbiota [[4], [5], [6]], enteral virus infections [7], and increased permeability of the intestine [6,8] have been linked to the autoimmune process leading to type 1 diabetes.

As a mechanistic hypothesis, gut dysbiosis and increased permeability may create a proinflammatory environment, interfering with healthy maturation of the gut immune system and leading to loss of tolerance. This hypothesis is supported by the findings that point to gut inflammation as a risk factor of other extraintestinal immune-mediated diseases such as rheumatoid arthritis, systemic lupus erythematosus, ankylosing spondyloarthritis, and allergic diseases [[9], [10], [11]].

Calprotectin and human β-defensin-2 (HBD-2) are considered markers of gut inflammation. Calprotectin, a leucocyte protein upregulated in the presence of neutrophil migration and activation, has been established in diagnostics and monitoring of inflammatory bowel disease [9]. HBD-2, an antimicrobial peptide secreted by enterocytes as part of mucosal defense, is less established but known to be induced by proinflammatory cytokines as well as microbial components [12].

Two studies have compared gut inflammation markers in children with islet autoantibody positivity and antibody-negative control children in the Finnish Dietary Intervention Trial for the Prevention of Type 1 Diabetes and Trial to Reduce Insulin dependent diabetes mellitus in the Genetically at Risk pilot studies [13,14]. de Goffau et al. [13] reported no difference in fecal calprotectin concentrations according to antibody status but did note higher calprotectin concentrations in children with higher genetic susceptibility to type 1 diabetes. Honkanen et al. [14] found no difference in calprotectin concentrations but reported higher fecal HBD-2 concentrations in autoantibody-positive children compared with autoantibody-negative control children, as well as in autoantibody-positive progressors from islet autoimmunity to type 1 diabetes compared with autoantibody-positive nonprogressors.

These earlier studies have, however, not been able to consider dietary factors, which are well-established modulators of gut microbiota composition and metabolism as well as systemic inflammation. Several dietary factors have also been associated with risk of islet autoimmunity and type 1 diabetes: gluten, cereals, and dietary fiber [15], as well as dairy products [[16], [17], [18]] have been associated with higher risk, whereas human milk feeding [16], cruciferous vegetables, berries [19], vitamin C [20], and n–3 (ω-3) fatty acids [[21], [22], [23]] have been associated with lower risk, although evidence remains mostly tentative.

The Finnish Type 1 Diabetes Prediction and Prevention (DIPP) cohort study aims to identify the environmental triggers of type 1 diabetes autoimmunity to reduce the global burden of disease. In this study, the primary objective was to assess whether elevated gut inflammation marker concentrations are associated with higher risk of islet autoimmunity in children with higher genetic risk of type 1 diabetes. In secondary analyses, we assessed whether gut inflammation marker levels are associated with dietary factors.

## Methods

### Study sample

The study sample was a nested case-control cohort sampled from the DIPP study. DIPP is an ongoing cohort study enrolling children with human leukocyte antigen (HLA)-conferred susceptibility to type 1 diabetes [24]. Children for the current study were sampled from DIPP participants born in Tampere and Oulu University Hospitals between 2 September, 1996 and 5 September, 2004, as this subcohort has detailed dietary data available on the total diet of the children during the first 6 y of life [25].

Children were screened for islet cell antibodies (ICAs) at 3, 6, 12, 18, and 24 mo and from thereafter annually until the age of 15 y. When ICAs were detected, all preceding and subsequent samples of the child were analyzed for biochemical markers of autoimmunity: antibodies against insulin (IAAs), glutamic acid decarboxylase (GADAs), and islet antigen-2 (IA-2As). For ICA screening, a standard indirect immunofluorescence method was applied. For IAA, GADA, and IA-2A determination, specific radiobinding assays were used [26].

Case children were defined as children who developed islet autoimmunity. Islet autoimmunity was defined as repeated positivity to ICAs and at least one biochemical marker of autoimmunity (ICA+1). The time of seroconversion to islet autoimmunity was defined as the time of the first ICA+1 positive sample. If the child developed clinical type 1 diabetes without prior ICA+1 positivity, they were also considered seroconverted to islet autoimmunity, and the time of islet autoimmunity was defined as the date of diagnosis of type 1 diabetes. Information on the diagnosis of type 1 diabetes was acquired from the Finnish Pediatric Diabetes Register [27].

In the nested case-control setting, 2 control children were randomly selected to match each of the case children by date of birth within 3 mo, sex, HLA genotype, and area of birth (Oulu and Tampere University Hospital areas). Additionally, case and control children had to come from different families, and control children should not have had repeated ICA+1 positivity or diabetes at the time of their matched cases confirmed islet autoimmunity [28,29].

The inclusion criteria for the present study were having ≥1 stool sample available at either of the study age points of 6 and 12 mo. Stool samples from case children had to be collected before seroconversion to islet autoimmunity. Case children needed to have ≥1 matched control child with a stool sample available at the same age point, the goal being 2.

**FIGURE 1:**
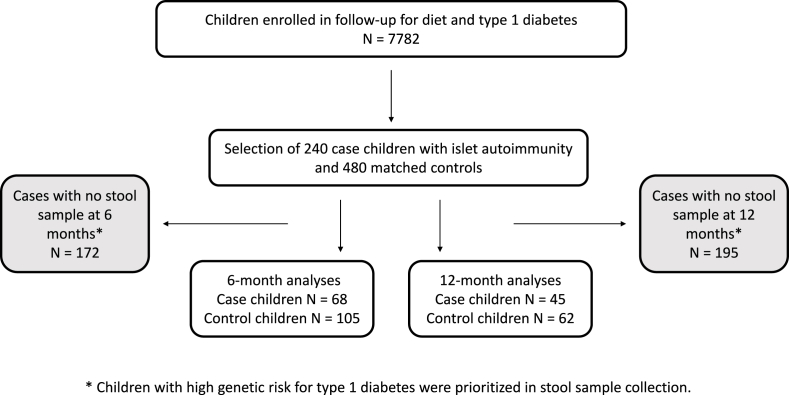
Flow chart of the nested case-control study within the Finnish Type 1 Diabetes Prediction and Prevention study (DIPP) cohort. Islet autoimmunity defined as repeated positivity for islet cell antibodies (ICA) and at least 1 biochemical autoantibody of the 3 that were analyzed (insulin autoantibodies [IAA], glutamic acid decarboxylase antibodies [GADA], or islet antigen-2 antibodies [IA-2A]) or presence of clinical type 1 diabetes. ∗Children with high genetic risk for type 1 diabetes were prioritized in stool sample collection.

These inclusion and exclusion criteria resulted in a sample of 163 individual children, of which 75 were case children and 88 control children. Mean age at the time of seroconversion to islet autoimmunity was 5.15 y (SD 3.49 y). Characteristics of the study children are presented in Table 1. Of the control children, 11 served as controls for 2 different case children, and 3 served as controls for 3 different case children. Additionally, 10 children served as controls first, but also developed islet autoimmunity later (range: 0.70–6.52 y after their matched case), and therefore served as both cases and controls. One of these 10 children served as a control for 2 cases. These duplications resulted in samples of 68 case children and 105 control children available for conditional logistic regression at 6 mo of age and 45 case children and 62 control children at 12 mo of age (Figure 1). The secondary analyses were not analyzed in the case-control design, and duplicate data of children present in >1 risk set were removed. This resulted in a sample of 153 individual children at 6 mo (147 with food records available) and 97 individual children at 12 mo (91 with food records available).

**TABLE 1:** Characteristics of the case children with islet autoimmunity and control children

Characteristic	Case children^1^ (*n* = 75)	Control children^2^^,^^3^ (*n* = 88)
	*n* (%)	*n* (%)
Sex		
Female	33 (44)	42 (48)
Male	42 (56)	46 (52)
Genetic risk group		
Moderate	4 (5)	4 (4.5)
High	71 (95)	84 (95.5)
Region		
Tampere	49 (65)	56 (64)
Oulu	26 (35)	32 (36)
First-degree relative with (any) diabetes		
No	63 (84)	78 (89)
Yes	11 (15)	8 (9)
Missing	1 (1)	2 (2)
Cow milk allergy^4^		
No	67 (89)	79 (90)
Yes	8 (11)	9 (10)
Human milk-fed ≥3 mo		
No	13 (17)	11 (12.5)
Yes	62 (83)	77 (87.5)
Human milk-fed ≥6 mo		
No	25 (33)	30 (34)
Yes	50 (67)	58 (66)

1 Islet autoimmunity defined as repeated positivity for islet cell antibodies and ≥1 biochemical autoantibody of the 3 that were analyzed (insulin autoantibodies [IAA], glutamic acid decarboxylase antibodies [GADA], or islet antigen-2 antibodies [IA-2A]) or presence of clinical type 1 diabetes.

2 Control children matched for date of birth within 3 mo, sex, human leukocyte antigen (HLA) genotype, and area of birth.

3 Eleven children served as controls for 2 cases and 3 children for 3 cases but were included only once in the total number of control children. Of the control children, 10 became a case at a later date and were counted as cases. Of these 10 children, 1 served as control for 2 cases.

4 Cow milk allergy by the age of 3 y.

Local ethics committees approved the DIPP study protocol. Informed consent was obtained from families participating in the study.

### Background variables

Background information on maternal and paternal vocational education, family history of diabetes of any type, and home municipality for urban/rural classification was collected with a questionnaire completed by the caretakers of the child at birth. The mother’s gestational weight gain was measured during standard maternal health clinic visits and later self-reported via a maternal questionnaire. Perinatal information on child’s sex, duration of gestation, hospital of birth, mode of delivery, birth length, birth weight, and maternal smoking during pregnancy was obtained directly from the medical birth registers of the university hospitals. Information on house pets before 5 y of age was collected through a separate allergy and asthma questionnaire completed by the caretakers of the child when the child was 5 y old. Information on cow milk allergy up to the age of 3 y was obtained from the registers of the Social Insurance Institution as well as inquired during the study visits at 6 mo and 1, 2, and 3 y. The length and weight of the child aged 6 and 12 mo were measured by trained study nurses at study visits. Ponderal index was calculated from child’s weight and length as kilograms divided per meters cubed (kg/m^3^).

### Dietary assessment

Three-day food records were collected from the children at 6 mo and 12 mo of age. Trained research nurses instructed the families in completing the food records and checked all records upon return. Trained nutritionists were responsible for entering the food record data into the nutritional calculation software Finessi. All food and nutrition calculations in Finessi are based on the Finnish national food composition database Fineli, which is regularly updated and has been supplemented with nutritional information of commercial infant foods sold in Finland. Dietary methods have been described in detail previously [25].

Information on duration of exclusive and overall human milk feeding as well as introduction to solid foods was collected with age-specific questionnaires when the children were 3, 6, 12, and 24 mo old, as well as through an “age at introduction of new foods” form continuously completed by the caretakers at home and checked by study nurses at each study visit [30].

### Nutrients and food groups

Nutrient intakes were assessed as the sum of intake from foods, human milk, and supplements. In addition to total dietary fiber, we assessed the fractions of insoluble dietary fiber, soluble dietary fiber that precipitates in 78% aqueous ethanol (high-molecular-weight-soluble dietary fiber), and soluble dietary fiber that remains soluble in 78% aqueous ethanol (nondigestible oligosaccharides) [31]. In this study, only dietary fiber from foods was considered; dietary fiber from supplements or human milk was not included.

Amount of human milk was calculated using estimated energy requirements based on child weight and the growth of the child and the energy intake from other foods based on the food records [32]. Definitions of food groups can be found in Supplemental Table 1.

### Stool samples

Stool samples were collected primarily from participants carrying the high-risk HLA genotype as part of the DIPP follow-up protocol. For this study, samples from ∼6 mo and 12 mo of age were used. In practice, samples could not always be acquired exactly at the set age points, so 6-mo-samples were collected from children aged 4–7 mo and the 12-mo-samples from children aged 9–14 mo.

### Analysis of fecal calprotectin and HBD-2 concentrations

Stool samples from the infants were collected by parents at home and sent to the laboratory using regular mail. Samples were stored at −80°C until analyzed. Fecal calprotectin and HBD-2 concentrations were analyzed from stool samples with commercial ELISA kits according to manufacturer’s instructions (Calpro AS; β-Defensin 2 ELISA Kit, Immundiagnostik). Briefly, ∼100 mg feces was obtained from each frozen sample. Extraction buffer was then added at a dilution of 1:50 for both HBD-2 and calprotectin. Fecal material with the extraction buffer was vortexed for 30 sec and mixing was continued in a shaker at 1000 rpm for 3 min or until solid particles had dissolved. Samples were then centrifuged for 10 min at 10,000 × *g* at room temperature, and the supernatants were collected and stored at −20°C until measured.

### Statistical methods

In the primary analysis, associations of fecal calprotectin and HBD-2 concentrations with the risk of islet autoimmunity were assessed by conditional logistic regression analysis. Conditional logistic regression allows accounting for the matching of cases and controls. Calprotectin and HBD-2 were tested in the conditional logistic regression model as both continuous and categorized variables (age group-specific tertiles). For HBD-2, the model showed better performance according to chi-square tested −2 log likelihood values when HBD-2 was entered into the model as categorized. For calprotectin, there was either no difference or the continuous variable showed slightly better performance according to the chi-square tested −2 log likelihood values. Therefore, results on HBD-2 are reported for tertiles and calprotectin as a continuous variable.

For sensitivity analysis of the primary analysis, the unadjusted analysis was carried out with and without children with cow milk allergy. This is because cow milk allergy in young children has gastrointestinal inflammatory manifestations including enteropathy, increased inflammatory cell infiltration, and cytotoxic expression [[33], [34], [35]]. Cow milk allergy is also associated with elevated fecal calprotectin concentrations [[36], [37], [38]] and has been linked with higher risk of type 1 diabetes [39].

Based on previous literature, maternal vocational education [40], breastfeeding status at 6 mo [16], and dietary fiber [15] and fat intake [21] were included as covariates for adjustment in the primary analyses. Energy-adjustment of both primary and secondary analyses was done in accordance with the multivariate nutrient density model [41] by dividing nutrient and food intake variables with total energy intake in megajoules and adding total energy intake as a covariate.

Correlation of calprotectin and HBD-2 with each other at 6 and 12 mo was assessed with Spearman’s correlation. Calprotectin and HBD-2 concentrations exhibited right-skewed distributions and were transformed to natural logarithmic scale for the remainder of the secondary analyses.

In the secondary analyses, associations of background and perinatal characteristics with fecal inflammation marker levels were assessed with independent samples *t* test or analysis of variance depending on the number of categories of the independent variable. Associations of exact age at stool sample collection, energy-adjusted food and nutrient intakes, human milk feeding history and status, and introduction to solid foods with fecal inflammation marker levels were assessed with the general linear model. All the general linear models were adjusted for case-control status, and all models including dietary factors were also adjusted for exact age at sample collection.

Food variables were used as either dichotomous, categorical, or continuous depending on the proportion of users within the assessed age group: dichotomous when the proportion of users in the age group was <50% (nonusers compared with users), categorical when the proportion of users was ≥50% but <75% (nonusers compared with those with energy-adjusted intake below the users’ median and those with energy-adjusted intake equal to or above the users’ median), and continuous when the proportion of users was ≥75%. For continuous independent variables, distribution was considered acceptable if residual plots of the model were determined as normally distributed based on visual examination.

Introduction to solid foods or infant formula variables were calculated based on the participant’s exact age at the time of the stool sample collection. Information on duration of human milk feeding and age of introduction to solid foods was collected from questionnaires that covered the whole first year of life.

The analyses were performed in IBM SPSS Statistics Version 29.0 (IBM Corporation). Missing data were handled by listwise deletion. For the primary outcome of islet autoimmunity, *P* values of <0.05 were considered significant. For the secondary analyses of nutrients, foods, human milk feeding history and status, and introduction to solid foods, the Benjamini-Hochberg method of false discovery rate (FDR) correction was applied, and adjusted *P* values of <0.1 were considered significant.

## Results

Characteristics of the study children are presented in Table 1. No noticeable differences were observed between the case and control children. Median absolute intakes of food groups and nutrients in the 6- and 12-mo age groups are presented in Supplemental Table 2.

### Gut inflammation marker levels and risk of islet autoimmunity

At 12 mo, HBD-2 concentration in the middle or highest tertiles compared with the lowest showed a borderline association with risk of islet autoimmunity in unadjusted analyses (Table 2). Calprotectin at 6 or 12 mo of age was not associated with later risk of islet autoimmunity (Table 2).

**TABLE 2:** Fecal calprotectin and HBD-2 concentrations at 6 and 12 mo of age and risk of later islet autoimmunity

	Unadjusted analysis^1^		Unadjusted sensitivity analysis: cow milk allergy^2^		Adjusted: maternal vocational education, human milk feeding, dietary factors^3^	
	OR (95% CI)	*P*-trend	OR (95% CI)	*P*-trend	OR (95% CI)	*P*-trend
Calprotectin, continuous (10 mg/kg)						
At 6 mo^4^	1.05 (0.99, 1.11)		1.09 (1.00, 1.19)		1.05 (0.98, 1.13)	
At 12 mo^5^	1.02 (0.93, 1.12)		1.00 (0.90, 1.12)		1.03 (0.92, 1.14)	
HBD-2, tertiles						
At 6 mo^4^		0.136		0.051		0.157
Middle vs. lowest	2.09 (1.01, 4.31)		2.51 (1.09, 5.79)		2.19 (0.97, 4.96)	
Highest vs. lowest	1.52 (0.71, 3.25)		2.56 (1.06, 6.22)		1.27 (0.53, 3.03)	
At 12 mo^5^		0.063		0.037		0.248
Middle vs. lowest	3.23 (1.03, 10.08)		6.72 (1.39, 32.61)		2.03 (0.59, 7.03)	
Highest vs. lowest	3.02 (1.05, 8.69)		4.49 (1.21, 16.62)		2.89 (0.78, 10.65)	

Abbreviations: CI, confidence interval; GADA, glutamic acid decarboxylase antibodies; HBD-2, human β-defensin-2; HLA, human leukocyte antigen; IA-2A, islet antigen-2 antibodies; IAA, insulin autoantibodies; OR, odds ratio.

The sample size of unadjusted analyses at 6 mo, *n* = 173 (case children 68, control children 105); at 12 mo, *n* = 107 (case children 45, control children 62). Statistical method: conditional logistic regression with case-control status as the dependent variable. Case children with islet autoimmunity defined as repeated positivity for islet cell antibodies and also positivity for ≥1 biochemical autoantibody of the 3 that were measured (IAA, GADA, or IA-2A) or presence of clinical type 1 diabetes. Control children matched for date of birth within 3 mo, sex, HLA genotype, and area of birth.

1 Model with no confounding factors.

2 Children with cow milk allergy by the age of 3 y and their matched pairs were excluded. Resulting sample sizes: 6 mo, *n* = 144 (61 cases, 83 controls); 12 mo, *n* = 91 (39 cases, 52 controls).

3 Maternal vocational education (none; vocational school or course; upper secondary vocational; academic), human milk feeding status at 6 mo of age (yes/no), total energy, energy-adjusted fat, and dietary fiber intake. Sample sizes resulting from missing values: 6 mo, *n* = 156 (63 cases, 93 controls), 12 mo, *n* = 94 (42 cases, 52 controls).

4 Samples representing 6 mo of age collected at 4–7 mo of age.

5 Samples representing 12 mo of age collected at 9–14 mo of age.

In sensitivity analyses, children with cow milk allergy were excluded from the analyses. The association of HBD-2 with risk of islet autoimmunity was strengthened at both age points, being significant at 12 mo (Table 2). There was also a borderline association between calprotectin concentration at 6 mo of age and risk of islet autoimmunity. Adjusting the analyses for maternal education, human milk feeding status at 6 mo of age, and dietary factors (energy, energy-adjusted fat, and dietary fiber intake) weakened the association of HBD-2 at 12 mo with risk of islet autoimmunity (Table 2).

### Nutrient and food intake and gut inflammation marker levels

Higher energy intake was associated with lower HBD-2 at 12 mo of age (Table 3). At 12 mo of age, higher energy-adjusted intake of total fat and different types of fatty acids (SFA, MUFA, PUFA, and n–6 PUFA) was associated with higher HBD-2 (Table 3). Higher energy-adjusted intake of carbohydrate, dietary fiber (total and insoluble), magnesium, and potassium was associated with lower HBD-2 (Table 3). No other associations of energy-adjusted nutrients (Table 3) or foods (Table 4) with gut inflammation marker levels were significant after FDR-correction.

**TABLE 3:** Associations of energy-adjusted intakes of nutrients with fecal HBD-2 and calprotectin concentrations at 6 and 12 mo of age

	6 mo^1^*n* = 147		12 mo^1^*n* = 91	
	Calprotectin	HBD-2	Calprotectin	HBD-2
	β (95% CI)	β (95% CI)	β (95% CI)	β (95% CI)
Energy, MJ	−0.390 (−0.831, 0.050)	−0.342 (−0.768, 0.083)	0.088 (−0.251, 0.428)	−0.442 (−0.776, −0.108)
Fat, g/MJ	0.094 (−0.018, 0.206)	0.099 (−0.009, 0.207)	0.133 (0.015, 0.250)	0.219 (0.110, 0.328)
SFA, g/MJ	0.198 (−0.025, 0.422)	0.191 (−0.025, 0.407)	0.258 (0.010, 0.506)	0.372 (0.135, 0.609)
MUFA, g/MJ	0.173 (−0.107, 0.452)	0.234 (−0.035, 0.503)	0.265 (0.009, 0.521)	0.444 (0.204, 0.683)
PUFA, g/MJ	0.590 (−0.134, 1.315)	0.415 (−0.288, 1.118)	0.491 (−0.011, 0.993)	0.665 (0.182, 1.148)
n–3, g/MJ	0.170 (−1.896, 2.237)	−0.796 (−2.790, 1.198)	1.789 (−0.482, 4.060)	1.972 (−0.251, 4.195)
n–6, g/MJ	0.719 (−0.080, 1.519)	0.649 (−0.124, 1.423)	0.541 (−0.048, 1.130)	0.778 (0.212, 1.344)
Protein, g/MJ	0.026 (−0.116, 0.168)	−0.060 (−0.197, 0.077)	−0.080 (−0.191, 0.031)	−0.124 (−0.231, −0.017)
Carbohydrate, g/MJ	−0.062 (−0.125, 0.001)	−0.048 (−0.109, 0.013)	−0.056 (−0.123, 0.012)	−0.086 (−0.151, −0.021)
Sucrose, g/MJ	−0.081 (−0.321, 0.158)	−0.226 (−0.454, 0.003)	−0.020 (−0.095, 0.055)	−0.041 (−0.114, 0.033)
Dietary fiber, g/MJ	−0.136 (−0.319, 0.048)	−0.159 (−0.336, 0.018)	−0.198 (−0.422, 0.027)	−0.294 (−0.510, −0.078)
IDF, g/MJ	−0.211 (−0.496, 0.074)	−0.268 (−0.542, 0.006)	−0.289 (−0.648, 0.070)	−0.457 (−0.801, −0.113)
SDFP, g/MJ	−0.412 (−0.957, 0.133)	−0.413 (−0.939, 0.114)	−0.643 (−1.353, 0.066)	−0.858 (−1.544, −0.172)
SDFS, g/MJ	−0.057 (−2.176, 2.062)	−0.223 (−2.270, 1.825)	−0.582 (−1.904, 0.739)	−0.936 (−2.225, 0.353)
Vitamins				
Vitamin A, μg/MJ	−0.001 (−0.003, 0.002)	−0.002 (−0.004, 0.001)	0.002 (−0.002, 0.006)	0.000 (−0.003, 0.004)
Vitamin B1, mg/MJ	−0.074 (−2.914, 2.766)	−0.376 (−3.120, 2.369)	0.186 (−4.079, 4.450)	−0.050 (−4.241, 4.140)
Vitamin B2, mg/MJ	−0.069 (−1.367, 1.229)	−0.185 (−1.439, 1.070)	−0.020 (−1.910, 1.871)	0.031 (−1.826, 1.889)
Vitamin B3, mg/MJ	0.169 (−0.106, 0.443)	−0.067 (−0.333, 0.199)	−0.093 (−0.510, 0.324)	−0.056 (−0.466, 0.355)
Vitamin B6, mg/MJ	0.084 (−1.932, 2.101)	−0.948 (−2.891, 0.994)	0.332 (−3.462, 4.125)	−0.028 (−3.756, 3.699)
Vitamin B12, μg/MJ	0.139 (−0.775, 1.054)	−0.114 (−0.998, 0.770)	−0.311 (−1.139, 0.517)	−0.445 (−1.255, 0.365)
Vitamin C, mg/MJ	−0.025 (−0.051, 0.000)	−0.009 (−0.034, 0.015)	0.008 (−0.017, 0.033)	0.009 (−0.016, 0.033)
Vitamin D, μg/MJ	−0.054 (−0.181, 0.073)	−0.023 (−0.146, 0.100)	0.064 (−0.115, 0.243)	−0.061 (−0.237, 0.115)
Vitamin E, mg/MJ	−0.197 (−0.555, 0.162)	−0.012 (−0.360, 0.335)	0.367 (0.028, 0.707)	0.229 (−0.110, 0.568)
Folate, μg/MJ	−0.014 (−0.040, 0.012)	−0.013 (−0.038, 0.012)	0.016 (−0.022, 0.055)	−0.021 (−0.058, 0.017)
Minerals				
Calcium, mg/MJ	−0.001 (−0.008, 0.006)	0.002 (−0.004, 0.009)	0.000 (−0.003, 0.004)	−0.002 (−0.005, 0.001)
Iron, mg/MJ	−0.152 (−0.333, 0.029)	−0.112 (−0.287, 0.064)	−0.151 (−0.399, 0.096)	0.090 (−0.163, 0.343)
Magnesium, mg/MJ	−0.017 (−0.039, 0.005)	−0.023 (−0.043, −0.002)	−0.021 (−0.045, 0.003)	−0.036 (−0.059, −0.014)
Potassium, mg/MJ	−0.002 (−0.003, 0.000)	−0.001 (−0.003, 0.000)	−0.001 (−0.003, 0.001)	−0.003 (−0.005, −0.001)
Selenium, μg/MJ	0.140 (−0.039, 0.318)	0.059 (−0.114, 0.233)	−0.192 (−0.345, −0.039)	−0.036 (−0.191, 0.120)
Zinc, mg/MJ	0.615 (−0.281, 1.511)	0.215 (−0.655, 1.086)	−0.306 (−1.022, 0.410)	−0.318 (−1.021, 0.385)

Abbreviations: CI, confidence interval; HBD-2, human-β-defensin-2; IDF, insoluble dietary fiber; MUFA, monounsaturated fatty acid; PUFA; polyunsaturated fatty acid; SDFP, soluble dietary fiber that precipitates in 78% aqueous ethanol; SDFS, soluble dietary fiber that remains soluble in 78% aqueous ethanol; SFA, saturated fatty acid.

Subgroups are indented. Statistical method: general linear model with natural logarithm transformed calprotectin/HBD-2 as dependent variable. Models controlled for case-control status, exact age at stool sample collection, and total energy intake. 1Only individual children with food intake data available were included in the analysis. This resulted in a sample of 147 individual children at 6 mo and 91 individual children at 12 mo.

**TABLE 4:** Associations of energy-adjusted intakes of food ingredients with fecal calprotectin and HBD-2 concentrations at 6 and 12 mo of age

	6 mo^1^*n* = 147			12 mo^1^*n* = 91		
	Intake group^2^	Calprotectin	HBD-2	Intake group^2^	Calprotectin	HBD-2
		β (95% CI)	β (95% CI)		β (95% CI)	β (95% CI)
Human milk	< median	−0.179 (−0.590, 0.232)	−0.121 (−0.519, 0.277)	users	−0.196 (−0.760, 0.367)	−0.183 (−0.737, 0.370)
	≥ median	−0.002 (−0.411, 0.407)	−0.046 (−0.442, 0.350)		—	—
Infant formula	< median	0.055 (−0.358, 0.468)	0.005 (−0.393, 0.403)	< median	0.152 (−0.285, 0.588)	−0.038 (−0.477, 0.401)
	≥ median	0.156 (−0.259, 0.571)	0.198 (−0.202, 0.599)	≥ median	0.528 (0.097, 0.960)	0.243 (−0.192, 0.677)
Cereals		−0.013 (−0.049, 0.024)	−0.035 (−0.070, 0.001)		−0.034 (−0.070, 0.001)	−0.035 (−0.069, 0.000)
Gluten containing	< median	−0.185 (−0.598, 0.228)	−0.187 (−0.588, 0.213)		−0.023 (−0.073, 0.027)	−0.048 (−0.096, 0.001)
	≥ median	0.146 (−0.262, 0.553)	−0.014 (−0.409, 0.382)		—	—
Wheat	< median	−0.117 (−0.531, 0.298)	−0.029 (−0.430, 0.371)		−0.030 (−0.102, 0.042)	−0.068 (−0.138, 0.002)
	≥ median	−0.097 (−0.514, 0.320)	−0.129 (−0.532, 0.274)		—	—
Rye	users	0.207 (−0.162, 0.576)	0.003 (−0.356, 0.361)		−0.044 (−0.162, 0.073)	−0.070 (−0.185, 0.045)
Non-gluten		−0.022 (−0.067, 0.022)	−0.041 (−0.083, 0.002)		−0.028 (−0.068, 0.012)	−0.012 (−0.052, 0.028)
Oats	< median	−0.198(−0.635, 0.239)	−0.112 (−0.529, 0,306)		−0.031 (−0.078, 0.016)	−0.019 (−0.066, 0.027)
	≥ median	−0.165 (−0.600, 0.270)	−0.410 (−0.826, 0.006)		—	—
Dairy		0.000 (−0.001, 0.002)	0.000 (−0.001, 0.002)		0.000 (−0.002, 0.003)	0.001 (−0.001, 0.004)
Non-fermented		0.000 (−0.001, 0.002)	0.000 (−0.001, 0.002)		0.001 (−0.002, 0.003)	0.002 (−0.001, 0.004)
Fermented	users	0.073 (−0.411, 0.557)	−0.108 (−0.575, 0.360)		−0.005 (−0.016, 0.006)	−0.006 (−0.017, 0.004)
Meat	< median	0.157 (−0.276, 0.590)	0.045 (−0.377, 0.467)		−0.033 (−0.068, 0.002)	0.014 (−0.021, 0.049)
	≥ median	0.330 (−0.101, 0.761)	0.024 (−0.395, 0.444)		—	—
Red meat	< median	0.281 (−0.130, 0.692)	0.094 (−0.306, 0.494)		−0.038 (−0.076, 0.000)	0.029 (−0.009, 0.066)
	≥ median	0.245 (−0.166, 0.655)	0.007 (−0.392, 0.406)		—	—
Fish	users	0.483 (−0.024, 0.989)	−0.117 (−0.612, 0.378)	users	−0.212 (−0.593, 0.169)	−0.039 (−0.416, 0.337)
Fruits and berries		−0.003 (−0.017, 0.011)	−0.007 (−0.021, 0.006)		−0.009 (−0.025, 0.008)	−0.002 (−0.018, 0.015)
Fruits		0.001 (−0.015, 0.017)	−0.008 (−0.024, 0.007)		−0.005 (−0.025, 0.015)	0.010 (−0.009, 0.029)
Berries	< median	−0.431 (−0.847, −0.015)	−0.056 (−0.463, 0.350)		−0.017 (−0.048, 0.014)	−0.031 (−0.061, −0.001)
	≥ median	−0.335 (−0.746, 0.077)	−0.245 (−0.647, 0.157)		—	—
Vegetables		−0.008 (−0.023, 0.007)	−0.010 (−0.025, 0.004)		−0.009 (−0.030, 0.013)	−0.010 (−0.031, 0.012)
Potato		−0.011 (−0.023, 0.001)	−0.006 (−0.018, 0.006)		−0.005 (−0.022, 0.012)	−0.017 (−0.033, 0.000)

Abbreviations: CI, confidence interval; HBD-2, human β-defensin-2.

Subgroups are indented. Statistical method: general linear model with natural logarithm transformed calprotectin/HBD-2 as dependent variable. Models controlled for case-control status, exact age at stool sample collection, and total energy intake. 1Only individual children with food intake data available included in analysis. This resulted in a sample of 147 individual children at 6 mo and 91 individual children at 12 mo. 2If proportion of users of food at age point ≥75%, energy-adjusted food intake used as continuous and no intake group specified. If proportion of users ≥50% and <75%, energy-adjusted food intake was used as a categorical variable with 3 categories (nonusers, intake < users’ median, intake ≥ users’ median). If the proportion of users <50%, food intake used as dichotomous (nonusers, users). Reference group for categorical variables in both cases: nonusers (not shown in table).

### Human milk feeding, introduction to solid foods, and gut inflammation marker levels

Children human milk-fed for <6 mo had higher HBD-2 concentrations at the time of the 12-mo stool sample collection than children human milk-fed for ≥6 mo (Table 5). No other differences in HBD-2 or calprotectin concentrations according to human milk feeding status or introduction to solid foods were found (Table 5, Table 6).

**TABLE 5:** Human milk feeding history, human milk feeding status at stool sample collection, and gut inflammation marker levels

	Yes	No	Missing	Calprotectin	HBD-2
	*n* (%)	*n* (%)	*n* (%)	β (95% CI)	β (95% CI)
6-mo stool sample (*n* = 153)					
Human milk-fed ≥ 3 mo	130 (85)	23 (15)	0 (0)	−0.058 (−0.529, 0.413)	−0.503 (−0.942, −0.065)
Human milk-fed at stool sample	101 (66)	49 (32)	3 (2)	0.020 (−0.342, 0.382)	−0.122 (−0.462, 0.217)
Exclusively human milk-fed at stool sample	3 (2)	148 (97)	2 (1)	NA	NA
12-mo stool sample (*n* = 97)					
Human milk-fed ≥ 3 mo	85 (88)	12 (12)	0 (0)	0.059 (−0.480, 0.597)	−0.689 (−1.276, −0.102)
Human milk-fed ≥ 6 mo	70 (72)	27 (28)	0 (0)	−0.026 (−0.420, 0.368)	−0.739 (−1.154, −0.325)
Human milk-fed at stool sample	24 (25)	72 (74)	1 (1)	0.141 (−0.272, 0.554)	−0.295 (−0.756, 0.165)
Exclusively human milk-fed at stool sample	0 (0)	96 (99)	1 (1)	NA	NA

Abbreviations: CI, confidence interval; HBD-2, human β-defensin-2.

Statistical method: general linear model with natural logarithm transformed calprotectin/HBD-2 as dependent factor. Models controlled for case-control status and exact age at stool sample collection.

**TABLE 6:** Introduction to foods and gut inflammation marker levels at the time of the 6-mo stool sample collection.

	Yes	No	Missing	Calprotectin	HBD-2
	*n* (%)	*n* (%)	*n* (%)	β (95% CI)	β (95% CI)
Introduced to infant formula	118 (77)	30 (20)	5 (3)	0.138 (−0.398, 0.456)	0.029 (−0.269, 0.545)
Introduced to dairy	130 (85)	21 (14)	2 (1)	0.123 (−0.356, 0.601)	0.112 (−0.346, 0.571)
Introduced to wheat, rye, barley, and/or oats	106 (69)	43 (28)	4 (3)	0.186 (−0.234, 0.607)	−0.420 (−0.811, −0.028)
Introduced to meat	107 (70)	41 (27)	5 (3)	−0.032 (−0.439, 0.374)	−0.072 (−0.459, 0.314)
Introduced to fish	34 (22)	107 (70)	12 (8)	0.327 (−0.074, 0.728)	−0.106 (−0.494, 0.282)
Introduced to fruits or berries	142 (93)	9 (6)	2 (1)	−0.343 (−1.103, 0.417)	−0.414 (−1.135, 0.306)
Introduced to root vegetables	145 (95)	6 (4)	2 (1)	−0.237 (−1.134, 0.659)	−0.722 (−1.566, 0.121)

Abbreviations: CI, confidence interval; HBD-2, human β-defensin-2.

*n* = 153 participants. Actual ages 4–7 mo. Statistical method: general linear model with natural logarithm transformed calprotectin/HBD-2 as dependent variable. Models controlled for case-control status and exact age at stool sample collection.

### Background, sociodemographic, and perinatal factors and gut inflammation marker levels

More extensive maternal education was associated with lower HBD-2 concentrations at 12 mo of age (Supplemental Table 3). Cow milk allergy was associated with higher calprotectin at 12 mo of age (Supplemental Table 3). Maternal smoking during pregnancy was associated with higher calprotectin at 12 mo of age, although at this age point, there were only 3 children with maternal smoking (Supplemental Table 3).

There were no associations of fecal inflammation markers with sex, mode of delivery, gestational age, ponderal index, or weight gain of the child at either age point (Supplemental Table 3). There were also no associations with paternal education, family history of diabetes of any type, mother’s gestational weight gain, indoor house pets, area of birth, or living environment (Supplemental Table 3).

Exact age at the time of stool sample collection was inversely associated with fecal calprotectin (β: −3.683; 95% CI: −6.519, −0.847) and HBD-2 (β: −6.087; 95% CI: −8.773, −3.402) in the 6-mo age group but not in the 12-mo age group (calprotectin β: −0.708; 95% CI: −3.024, 1.608; HBD-2 β: 1.428; 95% CI: −1.170, 4.025). Calprotectin and HBD-2 concentrations also correlated with each other at 6 mo of age (Spearman’s rho: 0.381, *P* < 0.001) but not at 12 mo of age (Spearman’s rho: 0.193, *P* = 0.059).

## Discussion

### Principal results

In our study assessing gut inflammation marker levels, diet, and risk of islet autoimmunity in children with HLA-conferred susceptibility to type 1 diabetes, we found a borderline association of higher HBD-2 concentrations in early childhood with higher risk of later islet autoimmunity. In secondary analyses we found several dietary factors to be associated with fecal HBD-2 concentrations. Particularly, dietary fat intake was associated with higher HBD-2 concentrations and intake of dietary fiber, potassium, and magnesium with lower HBD-2 concentrations at 12 mo of age.

The borderline association of higher HBD-2 concentrations with higher risk of islet autoimmunity strengthened when children with cow milk allergy were excluded and weakened when adjusted for dietary factors and maternal education, and therefore should be interpreted with caution. Some of these changes may have been caused by reductions in sample size. However, a role for cow milk allergy is mechanistically plausible, as cow milk allergy in young children is associated with disturbances in the immune function of the intestine [[33], [34], [35], [36], [37], [38]] as well as higher risk of type 1 diabetes [39]. Furthermore, in a previous study, Honkanen et al. [14] reported higher HBD-2 concentrations in islet autoantibody-positive children compared with islet autoantibody-negative control children, as well as antibody-positive progressors to type 1 diabetes compared with antibody-positive nonprogressors. The possible association of higher HBD-2 with higher risk of autoimmunity we noted is in line with these results.

We found dietary factors to be associated with gut inflammation markers. Energy-adjusted carbohydrate, dietary fiber, magnesium, and potassium were nutrients associated with lower HBD-2 concentrations. Energy-adjusted fat and saturated and unsaturated fatty acids were associated with higher HBD-2 concentrations. Other weak associations were noted, but they were no longer significant after FDR-correction.

Our findings regarding associations of diet with inflammation are partly in line with those of Koivusaari et al. [42], who studied the associations of foods with fecal HBD-2 and calprotectin during the first year of life in a separate sample of 73 Finnish children with HLA-conferred susceptibility to type 1 diabetes. Koivusaari et al. [42] reported associations of fruits and juices, vegetables, and oats with lower HBD-2, which could be reflected by our finding of higher intake of dietary fiber and carbohydrates in association with lower HBD-2. However, Koivusaari et al. [42] also reported lower calprotectin in association with fruits and juices, vegetables, and potatoes, and higher calprotectin in association to human milk, which we did not find. The analyses by Koivusaari et al. [42] were longitudinal, and there were several differences in the dietary habits of the study populations that could explain some of the differences in findings. For example, a smaller proportion of children in the present study was human milk-fed, and the children consumed more potatoes, meat, and cereals than those in the study by Koivusaari et al. [42].

To our knowledge, no previous studies have assessed nutrient intake in association with gut inflammation markers. There are a limited number of previous studies in healthy children that have assessed differences in calprotectin or HBD-2 between infant formula and human milk-fed infants, and the results have been inconsistent [[43], [44], [45], [46]]. We found children that had stopped receiving human milk before 6 mo of age exhibited higher HBD-2 concentrations at 12 mo of age but found no associations between human milk feeding and inflammation markers if these factors were assessed at the same time point.

### Strengths and limitations

Due to the nested case-control setting of our study, we were able to assess gut inflammation marker levels before seroconversion to islet autoimmunity, whereas previous studies have been retrospective in design with fecal samples collected after the determination of islet cell antibody status [13,14]. Furthermore, we were able to use high quality food consumption data [47,48] to assess the relationship of dietary factors with gut inflammation marker levels.

Limitations in this study include a restricted sample size possibly resulting in a lack of power, the fact that stool samples were not always collected at the same time as the 3-d food records, not being able to include enteral infections or antibiotics use as confounding factors, and lack of information on timing and duration of cow milk allergy. Both bacterial and viral enteral infections are known to cause elevated calprotectin concentrations [9]. Assessing food consumption and especially the amount of human milk consumed is challenging, and although our assessment methods, study protocol, and database underwent a high level of quality control, some degree of error and confounding might still affect any findings regarding the results involving dietary intake.

We are unable to determine causality or shed light on the mechanisms of the observed associations due to our observational study design.

### Meaning of the study

From previous studies, it is known that gut inflammation markers are higher in infants than healthy adults and decrease with age [11,43,44,[49], [50], [51], [52]]. In our study, despite a larger sample size in the 6-mo age group, we observed stronger associations between diet, gut inflammation markers, and risk of autoimmunity at 12 mo of age than at 6 mo. At 6 mo of age, diet may not yet be as important of a factor for inflammation as it is in older age. Inflammation levels in younger infants may be determined by other factors such as genotype or microbiota.

Interestingly, we noted gut inflammation markers to have mostly inverse associations with dietary factors, apart from fats. Based on the foods and nutrients associated with lower gut inflammation marker levels, being introduced to solid foods (sources of dietary fiber, carbohydrate, and minerals) could stimulate barrier function, development of immune tolerance, or maturation of the gut microbiome, promoting the natural age-related decrease in gut inflammation markers. The gut microbiome is known to undergo compositional changes in association with the progression of complementary feeding and decreasing human milk intake [53,54], and in turn, the microbiota may mediate both local and systemic effects [[55], [56], [57], [58]]. However, evaluating the harm or benefit of associations of diet with the inflammatory markers is complicated, as there is no consensus on reference values for calprotectin or HBD-2 in infancy.

We noted HBD-2 to exhibit more associations with dietary factors than calprotectin. This could suggest HBD-2 is a more sensitive marker for the direct effects of nondigestible dietary components or microbiota on intestinal epithelia, as HBD-2 is produced locally by intestinal epithelial cells [12]. HBD-2 has been less commonly used in previous studies than the clinically more established calprotectin, but our findings could support adding HBD-2 as a marker in future studies assessing the effects of diet on intestinal inflammation, immunity, or microbiota.

The clinical significance of our findings remains unclear. Higher HBD-2 concentration at 1 y of age was associated with roughly a 3-fold risk of later islet autoimmunity, but this should be interpreted with caution due to the possible confounding. Our study does, however, suggest that even very early gut immune system function could play an important and long-lasting role in the process of type 1 diabetes-related autoimmunity, and more research should aim to shed light on such mechanisms. Furthermore, our study raises HBD-2 as a potentially sensitive marker of the inflammatory status of the infant gut and suggests several nutrients of interest to be taken into account in future studies assessing gut inflammation.

### Generalizability

The DIPP birth cohort consists of children with HLA-conferred susceptibility to type 1 diabetes, limiting the generalizability of our results to the general population. Additionally, children with high-risk HLA genotypes were prioritized in stool sampling, further limiting the representativeness of the current study sample. The secondary analyses of dietary factors with gut inflammation marker levels can be considered exploratory due to the large number of hypotheses tested. Furthermore, case-control sampling for the primary outcome of islet autoimmunity may have introduced bias to the secondary analyses. Our findings will therefore need to be confirmed by future studies.

### Conclusions

Higher HBD-2 concentrations in infancy may be associated with higher risk of islet autoimmunity. Dietary factors play a role in the inflammatory status of the infant gut. More insight is needed into the mechanisms and clinical significance of these associations.

## Author contributions

The authors’ responsibilities were as follows – JI, JT, RV, MK: designed the DIPP study; HH: designed the microbial and SMV the nutrition study within DIPP; TEIS, LH, SN, HH, MK, SMV: designed the current study; HMT: was responsible for data management and supervision of statistical analysis; MK: was responsible for the gut inflammation marker analyses; RV, MK: were responsible for antibody measurements and type 1 diabetes data; SA: supervised dietary data collection and processing, food composition database work, and dietary calculations; LP: was responsible for organizing DIPP stool sample collection and processing for the study; TEIS: performed statistical analysis; TEIS, LH, SN, SMV: wrote paper; SMV: had primary responsibility for the final content; and all authors read, critically revised, and approved the final manuscript.

### Conflict of interest

The authors report no conflicts of interest.

## Funding

Supported by the Academy of Finland grants 63672, 79685, 79686, 80846, 201988, 210632, 129492, 126813, 276475, and 339922 (to SMV); the Yrjö Jahnsson Foundation (to SMV); the Juho Vainio Foundation (to SMV); the Competitive Research Funding of the Tampere University Hospital grants 9E082, 9F089, 9G087, 9H092, 9J147, 9K149, 9L035, 9L117, 9M029, 9M114, 9N086, 9P017, 9P057, 9R012, 9R055, 9S015, 9S074, 9T072, 9U016, 9U065, 9V012, 9V072, 9X062, 9AA084, 9AB083, and 9AC099 (to SMV); Medical Research Funds of Turku (to JI and JT) and Oulu (to RV) University Hospitals; the European Foundation for the Study of Diabetes (supported by EFSD/JDRF/Lilly) (to SMV and HH); the Juvenile Diabetes Research Foundation grants 197032, 4-1998-274, 4-1999-731, 4-2001-435, 1-SRA-2016-342-M-R, and 1-41 SRA-2019-732-M-B (to JT, MK, RV), the Novo Nordisk Foundation and EU Biomed 2 grant BMH4-CT98-3314 (to JT, MK, and RV); Sigrid Jusélius Foundation (to JI, JT, MK, RV, and HH), and Doctoral Programs for Public Health (to SMV). The supporting sources were not involved in the study design, collection, analysis, and interpretation of data, writing of the report, or restrictions regarding publication.

## Data availability

Data described in the article will be made available upon reasonable request to the DIPP study data manager HMT pending application and approval. Analytic code will be available from the corresponding author upon reasonable request.
