# Supplementary material for: Gut Inflammation Markers, Diet, and Risk of Islet Autoimmunity in Finnish Children – A Nested Case-Control Study
Source: J Nutr. 2024 May 23;154(7):2244–54. doi: 10.1016/j.tjnut.2024.05.015 (PMC11282497; doi:10.1016/j.tjnut.2024.05.015)
Supplement: Multimedia component 1 [file mmc1.docx]

**Supplemental Table 1.** Definitions of food groups. Subgroups indented.

| Food group | Included | Not included |
| --- | --- | --- |
| Human milk |  |  |
| Infant formula | Cow’s milk and non-cow’s milk based infant formulas |  |
| Cereals | All cereals, starches in dry weight |  |
| Gluten containing cereals | Wheat, rye, barley in dry weight | Gluten-free starches |
| Wheat | Wheat in dry weight | Gluten-free wheat starch |
| Rye | Rye in dry weight |  |
| Non-gluten cereals | Oat, rice, corn, other non-gluten containing cereals, gluten-free starches in dry weight |  |
| Oat | Oat, oat in oat-based products (oat drink etc.) in dry weight |  |
| Dairy | All cow’s milk products |  |
| Non-fermented dairy | Cow’s milk based infant formula, milk, cream, ice cream, milk powder |  |
| Fermented dairy | Fermented milk and cream products, cheese |  |
| Meat | Red, white, and processed meat | Fish, seafood |
| Red meat | Beef, lamb, pork, offal, sausages (some sausages may contain poultry) | Poultry |
| Fish | Fish, seafood |  |
| Fruits and berries | Unprocessed and processed fruits and berries | Juices |
| Fruits | Unprocessed and processed fruits | Juices |
| Berries | Unprocessed and processed berries | Juices |
| Vegetables | Unprocessed and processed vegetables and edible fungi | Fruits, berries, juices, potato, legumes, nuts, seeds |
| Potato | Unprocessed and processed potatoes |  |

**Supplemental Table 2.** Proportion of users of food groups, and daily absolute intakes of food groups and nutrients at the age points 6 months and 12 months. Subgroups indented.

|  | | 6 months  *n* = 147 | | | | | 12 months  *n* = 91 | | |  | 6 months  *n* = 147 | | 12 months  *n* = 91 | |
| --- | --- | --- | --- | --- | --- | --- | --- | --- | --- | --- | --- | --- | --- | --- |
|  | Users % | | Median | | (IQR) | Users % | | Median | (IQR) |  | Median | (IQR) | Median | (IQR) |
| Human milk, g | | 61 | 370.7 | (0.0-701.9) | | | 13 | 0.0 | (0.0-0.0) | Energy, MJ | 2.85 | (2.63-3.09) | 3.69 | (3.22-4.13) |
| Infant formula, g | | 60 | 59.6 | (0.0-516.7) | | | 63 | 126.7 | (0.0-423.3) | Fat, g | 27.72 | (24.52-32.06) | 27.47 | (23.45-33.59) |
| Cereals, g | | 99 | 17.6 | (11.3-27.1) | | | 100 | 51.8 | (41.5-72.3) | SFA, g | 11.07 | (9.31-12.89) | 11.35 | (9.22-13.02) |
| Gluten containing, g | | 59 | 2.0 | (0.0-5.8) | | | 96 | 19.2 | (11.0-29.2) | MUFA, g | 10.60 | (9.32-12.05) | 9.59 | (7.87-12.03) |
| Wheat, g | | 56 | 0.8 | (0.0-3.5) | | | 95 | 11.5 | (6.3-19.3) | PUFA, g | 4.60 | (4.12-5.25) | 4.49 | (3.53-5.83) |
| Rye, g | | 32 | 0.0 | (0.0-0.8) | | | 81 | 3.8 | (1.1-7.2) | n-3, g | 0.84 | (0.70-1.01) | 0.81 | (0.60-0.97) |
| Non-gluten, g | | 98 | 14.4 | (9.0-23.3) | | | 100 | 32.3 | (21.8-48.4) | n-6, g | 3.69 | (3.25-4.15) | 3.59 | (2.66-4.79) |
| Oat, g | | 74 | 3.7 | (0.0-10.0) | | | 93 | 14.0 | (6.7-27.3) | Protein, g | 14.81 | (12.69-17.54) | 31.38 | (25.69-36.25) |
| Dairy, g | | 79 | 181.8 | (30.7-635.9) | | | 96 | 602.1 | (401.1-792.8) | Carbohydrate, g | 89.00 | (80.51-96.88) | 120.57 | (105.31-131.47) |
| Non-fermented, g | | 79 | 181.5 | (22.6-635.9) | | | 96 | 525.0 | (313.3-715.3) | Sucrose, g | 2.44 | (1.35-3.64) | 8.87 | (5.85-16.51) |
| Fermented, g | | 15 | 0.0 | (0.0-0.0) | | | 85 | 59.8 | (6.7-103.8) | Dietary fiber, g | 4.32 | (2.59-6.11) | 9.66 | (8.21-12.87) |
| Meat, g | | 74 | 9.7 | (0.0-18.8) | | | 100 | 41.6 | (22.9-53.4) | IDF, g | 2.67 | (1.59-3.78) | 6.02 | (4.99-7.46) |
| Red meat, g | | 65 | 7.0 | (0.0-14.4) | | | 95 | 30.8 | (14.3-39.6) | SDFP, g | 1.36 | 0.84-2.19) | 3.06 | (2.41-3.99) |
| Fish, g | | 13 | 0.0 | (0.0-0.0) | | | 40 | 3.5 | (0.0-5.9) | SDFS, g | 0.17 | (0.07-0.34) | 0.84 | (0.54-1.08) |
| Fruits and berries, g | | 97 | 35.1 | (21.3-57.5) | | | 99 | 63.4 | (33.4-91.9) | Vitamin A, µg | 569.71 | (480.86-662.21) | 442.52 | (348.04-570.38) |
| Fruits, g | | 94 | 28.7 | (11.8-49.7) | | | 97 | 42.8 | (25.9-65.6) | Vitamin B1, mg | 0.46 | (0.31-0.63) | 0.67 | (0.56-0.81) |
| Berries, g | | 67 | 5.9 | (0.0-16.5) | | | 84 | 11.8 | (2.0-27.8) | Vitamin B2, mg | 0.57 | (0.42-1.04) | 1.21 | (0.92-1.51) |
| Vegetables, g | | 96 | 31.0 | (14.7-49.3) | | | 100 | 49.8 | (34.3-71.8) | Vitamin B3, mg | 6.04 | (4.85-7.76) | 11.11 | (10.29-12.62) |
| Potato, g | | 95 | 32.2 | (16.8-62.8) | | | 100 | 67.0 | (44.7-94.4) | Vitamin B6, mg | 0.54 | (0.31-0.74) | 0.94 | (0.82-1.11) |
|  | |  |  |  | | |  |  |  | Folate, µg | 74.72 | (61.75-91.92) | 102.51 | (85.74-119.67) |
|  | |  |  |  | | |  |  |  | Vitamin B12, µg | 0.91 | (0.57-1.47) | 2.11 | (1.81-2.98) |
|  | |  |  |  | | |  |  |  | Vitamin C, mg | 79.24 | (69.06-95.85) | 72.64 | (52.38-95.94) |
|  | |  |  |  | | |  |  |  | Vitamin D, µg | 11.89 | (10.27-15.54) | 12.57 | (10.07-14.92) |
|  | |  |  |  | | |  |  |  | Vitamin E, mg | 4.36 | (3.63-5.72) | 4.45 | (2.98-6.12) |
|  | |  |  |  | | |  |  |  | Calcium, mg | 300.92 | (250.20-373.27) | 568.78 | (433.04-796.39) |
|  | |  |  |  | | |  |  |  | Iron, mg | 3.98 | (2.42-5.94) | 5.83 | (4.31-7.60) |
|  | |  |  |  | | |  |  |  | Magnesium, mg | 75.04 | (57.07-93.01) | 152.80 | (126.57-179.94) |
|  | |  |  |  | | |  |  |  | Potassium, mg | 938.73 | (756.68-1117.06) | 1746.08 | (1466.08-2037.77) |
|  | |  |  |  | | |  |  |  | Selenium, µg | 10.50 | (8.79-11.90) | 19.92 | (16.29-24.06) |
|  | |  |  |  | | |  |  |  | Zinc, mg | 3.86 | (3.40-4.35) | 5.34 | (4.70-6.29) |

Vitamin B3: niacin equivalents
Vitamin B6: pyridoxine vitamers
IDF: insoluble dietary fiber; SDFP: soluble dietary fiber that precipitates in 78% aqueous ethanol; SDFS: soluble dietary fiber that remains soluble in 78% aqueous ethanol; dietary fibre from foods only (not supplements or human milk)

**Supplemental Table 3.** Differences in natural logarithm transformed fecal human-β-defensin-2 (HBD-2) and calprotectin levels at 6 and 12 months of age by background and perinatal characteristics.

|  | 6 months^1^  N = 153 | | | | | 12 months^1^  N = 97 | | | | |
| --- | --- | --- | --- | --- | --- | --- | --- | --- | --- | --- |
|  |  | Calprotectin | | HBD-2 | |  | Calprotectin | | HBD-2 | |
|  | N | Mean (SD) | *P* | Mean (SD) | *P* | N | Mean (SD) | *P* | Mean (SD) | *P* |
| Sex |  |  | 0.364 |  | 0.677 |  |  | 0.299 |  | 0.140 |
| Female | 71 | 3.26 (1.09) |  | 3.85 (0.98) |  | 43 | 3.25 (0.96) |  | 3.65 (0.70) |  |
| Male | 82 | 3.10 (1.03) |  | 3.92 (1.10) |  | 54 | 3.07 (0.78) |  | 3.94 (1.15) |  |
| Case-control status |  |  | 0.514 |  | 0.646 |  |  | 0.755 |  | 0.110 |
| Case | 68 | 3.23 (1.11) |  | 3.92 (1.05) |  | 45 | 3.12 (0.94) |  | 3.98 (0.92) |  |
| Control | 85 | 3.12 (1.02) |  | 3.85 (1.05) |  | 52 | 3.17 (0.80) |  | 3.66 (1.02) |  |
| First degree relative with (any) diabetes |  |  | 0.663 |  | 0.571 |  |  | 0.165 |  | 0.124 |
| Yes | 18 | 3.28 (1.32) |  | 3.80 (0.70) |  | 10 | 2.80 (0.71) |  | 3.39 (0.85) |  |
| No | 132 | 3.17 (1.03) |  | 3.91 (1.09) |  | 86 | 3.20 (0.87) |  | 3.88 (0.97) |  |
| Missing | 3 |  |  |  |  | 1 |  |  |  |  |
| Maternal vocational education |  |  | 0.673 |  | 0.759 |  |  | 0.841 |  | 0.024 |
| None, vocational school, or course | 43 | 3.28 (1.08) |  | 3.97 (1.16) |  | 24 | 3.09 (0.76) |  | 4.26 (1.16) |  |
| Upper secondary vocational | 68 | 3.18 (1.03) |  | 3.90 (1.02) |  | 48 | 3.13 (0.85) |  | 3.69 (0.73) |  |
| Academic | 39 | 3.07 (1.11) |  | 3.80 (0.99) |  | 23 | 3.23 (1.01) |  | 3.59 (0.99) |  |
| Missing | 3 |  |  |  |  | 2 |  |  |  |  |
| Paternal vocational education |  |  | 0.372 |  | 0.675 |  |  | 0.626 |  | 0.103 |
| None, vocational school, or course | 68 | 3.06 (0.98) |  | 3.87 (1.05) |  | 41 | 3.23 (0.91) |  | 3.98 (0.97) |  |
| Upper secondary vocational | 49 | 3.34 (1.20) |  | 3.83 (1.05) |  | 33 | 3.06 (0.68) |  | 3.92 (0.97) |  |
| Academic | 31 | 3.14 (1.00) |  | 4.04 (1.10) |  | 21 | 3.06 (0.94) |  | 3.44 (0.91) |  |
| Missing | 5 |  |  |  |  | 2 |  |  |  |  |
| Maternal smoking during pregnancy |  |  | 0.934 |  | 0.810 |  |  | < 0.001 |  | 0.985 |
| Yes | 8 | 3.15 (1.37) |  | 3.78 (0.75) |  | 3 | 4.80 (0.54) |  | 3.82 (1.00) |  |
| No | 143 | 3.18 (1.05) |  | 3.87 (1.05) |  | 91 | 3.09 (0.83) |  | 3.80 (1.00) |  |
| Missing | 2 |  |  |  |  | 3 |  |  |  |  |
| Mode of delivery |  |  | 0.725 |  | 0.681 |  |  | 0.897 |  | 0.420 |
| Vaginal | 138 | 3.18 (1.08) |  | 3.88 (1.04) |  | 87 | 3.16 (0.83) |  | 3.82 (1.01) |  |
| Cesarean section | 14 | 3.08 (0.88) |  | 3.76 (0.98) |  | 9 | 3.12 (1.19) |  | 3.54 (0.62) |  |
| Missing | 1 |  |  |  |  | 1 |  |  |  |  |
| Mother’s gestational weight gain |  |  | 0.491 |  | 0.209 |  |  | 0.357 |  | 0.780 |
| 1^st^ quartile | 34 | 3.38 (1.04) |  | 4.04 (1.21) |  | 26 | 3.27 (0.86) |  | 3.90 (1.22) |  |
| 2^nd^ quartile | 34 | 3.15 (0.85) |  | 3.82 (0.89) |  | 21 | 3.33 (1.02) |  | 3.63 (0.67) |  |
| 3^rd^ quartile | 36 | 3.01 (1.27) |  | 4.09 (1.18) |  | 24 | 2.95 (0.86) |  | 3.84 (1.19) |  |
| 4^th^ quartile | 34 | 3.05 (1.12) |  | 3.61 (0.87) |  | 15 | 2.95 (0.74) |  | 3.94 (0.70) |  |
| Missing | 15 |  |  |  |  | 11 |  |  |  |  |
| Gestational age |  |  | 0.888 |  | 0.230 |  |  | 0.118 |  | 0.317 |
| Pre-term (under 259 days) | 11 | 3.22 (1.20) |  | 3.51 (0.79) |  | 7 | 3.34 (0.21) |  | 3.44 (0.77) |  |
| Full-term (259 days and over) | 141 | 3.17 (1.06) |  | 3.90 (1.04) |  | 89 | 3.14 (0.90) |  | 3.83 (1.00) |  |
| Missing | 1 |  |  |  |  | 1 |  |  |  |  |
| Ponderal index at birth |  |  | 0.808 |  | 0.378 |  |  | 0.256 |  | 0.654 |
| 1^st^ quartile | 38 | 3.09 (1.03) |  | 3.72 (1.03) |  | 26 | 3.06 (0.85) |  | 3.68 (0.93) |  |
| 2^nd^ quartile | 37 | 3.28 (1.10) |  | 3.88 (0.99) |  | 24 | 2.95 (0.80) |  | 3.69 (0.85) |  |
| 3^rd^ quartile | 38 | 3.08 (1.14) |  | 3.77 (0.98) |  | 23 | 3.20 (0.94) |  | 3.76 (1.00) |  |
| 4^th^ quartile | 38 | 3.24 (1.02) |  | 4.10 (1.12) |  | 22 | 3.44 (0.86) |  | 4.00 (1.11) |  |
| Missing | 2 |  |  |  |  | 2 |  |  |  |  |
| Ponderal index at age point |  |  | 0.132 |  | 0.051 |  |  | 0.151 |  | 0.264 |
| 1^st^ quartile | 38 | 3.03 (1.11) |  | 3.73 (1.00) |  | 23 | 2.87 (0.76) |  | 3.57 (0.96) |  |
| 2^nd^ quartile | 37 | 3.41 (1.15) |  | 4.26 (1.09) |  | 24 | 3.44 (0.85) |  | 4.01 (0.90) |  |
| 3^rd^ quartile | 36 | 3.23 (0.94) |  | 3.90 (1.02) |  | 23 | 3.25 (0.82) |  | 3.98 (1.00) |  |
| 4^th^ quartile | 38 | 2.89 (0.81) |  | 3.64 (0.99) |  | 24 | 3.10 (0.99) |  | 3.61 (1.09) |  |
| Missing | 4 |  |  |  |  | 3 |  |  |  |  |
| Indoor house pet during 1^st^ year of life |  |  | 0.397 |  | 0.576 |  |  | 0.409 |  | 0.825 |
| Yes | 38 | 3.23 (0.76) |  | 3.74 (0.91) |  | 31 | 3.22 (0.75) |  | 3.72 (0.94) |  |
| No | 75 | 3.11 (1.16) |  | 3.90 (1.01) |  | 45 | 3.06 (0.87) |  | 3.78 (1.03) |  |
| Missing | 40 |  |  |  |  | 21 |  |  |  |  |
| Living environment |  |  | 0.128 |  | 0.335 |  |  | 0.142 |  | 0.868 |
| Urban | 121 | 3.21 (1.10) |  | 3.85 (1.06) |  | 76 | 3.12 (0.83) |  | 3.82 (0.99) |  |
| Semi-urban | 11 | 3.51 (0.85) |  | 3.56 (0.55) |  | 9 | 3.68 (1.21) |  | 3.74 (0.96) |  |
| Rural | 20 | 2.77 (0.85) |  | 4.12 (1.04) |  | 11 | 2.99 (0.68) |  | 3.66 (1.00) |  |
| Missing | 1 |  |  |  |  | 1 |  |  |  |  |
| Cow’s milk allergy by 3 years of age |  |  | 0.956 |  | 0.849 |  |  | 0.009 |  | 0.339 |
| No | 136 | 3.17 (1.05) |  | 3.89 (1.06) |  | 89 | 3.08 (0.83) |  | 3.84 (1.01) |  |
| Yes | 17 | 3.16 (1.15) |  | 3.84 (0.94) |  | 8 | 3.91 (0.94) |  | 3.49 (0.66) |  |

Statistical method: two-tailed independent samples t-test or univariate analysis of variance with natural logarithm transformed calprotectin/human-β-defensin-2 (HBD-2) as dependent variable.
^1^ Only individual children included, meaning children serving as a control for more than one case child included only once, and children serving as both case and control included only once and counted as cases. This resulted in a sample of 153 individual children at 6 months, and 97 individual children at 12 months.
